# Success factors for adherence in hyposensitization 

**DOI:** 10.5414/ALX01430E

**Published:** 2018-09-01

**Authors:** N. Sondermann, K. Shah-Hosseini, K. Henkel, A. Schwalfenberg, R. Mösges

**Affiliations:** 1 Institut für medizinische Statistik, Informatik und Epidemiologie, Universitätsklinikum Köln,; 2 Deutscher Allergie- und Asthmabund e.V., Mönchengladbach

**Keywords:** hyposensitization, adherence, treatment compliance, SLIT (sublingual immunotherapy), SCIT (subcutaneous immunotherapy)

## Abstract

For the success of an immunotherapy regimen, adherence is a major success factor. The goal of our study was to identify the factors that positively and negatively influence patient compliance, and to create strategies to improve it. Four questionnaires were designed for different patient groups: A – after immunotherapy; B – during immunotherapy; C – before immunotherapy; D – no experience with immunotherapy. From March to October 2008, 790 questionnaires were collected. For the first group, questionnaire A was answered by 272 patients. Of these, 15.8% had dropped out of immunotherapy. Women had higher dropout rates than men (16.8% vs. 12.3%). The following aspects of immunotherapy were viewed by the patients as negative: time consuming (69.5%), adverse reactions (62.5%), insufficient patient information (53.7%), no change in use of symptomatic medication (33.8%) and no change in symptoms (60.7%). Despite the mentioned drawbacks, 74% of all patients would still recommend allergen immunotherapy. Questionnaire B was completed by 281 patients. In this group, 8.7% had already considered dropping out. The following unfavourable aspects were identified: time consuming (66.2%), adverse reactions (61.9%), insufficient patient information (54.8%), no change in symptoms (51.2%) and use of symptomatic medication (47.0%). Despite this, up to 95.4% of all patients would recommend immunotherapy. Questionnaire C was filled-out by 55 patients. The following reasons were rated by the patients as “important” or “very important” for the decision to start hyposensitization: long-lasting symptom alleviation (100%), few adverse reactions (98.2%), comprehensive patient information (96.3%), easy integration into daily routine (89.1%), re-assessment of therapy by doctor (83.3%) and reduced need for symptomatic medication (81.8%). Questionnaire D was filled in by 182 participants. 89% had already heard the term hyposensitization before. Their general knowledge regarding this therapy was average (3.23 on a scale of 1 – 6; where 1 = optimum). Long-lasting symptom alleviation (99.5%), comprehensive patient information (97.8%), easy integration into daily routine (96.1%), reduced symptomatic medication use (92.6%) and re-assessment by doctor (88.8%) were considered “very important” or “important” characters in the desired immunotherapy regime. Adherence to the hyposensitization schedule is essential for its success. The treating doctor should aim at choosing the right therapy and working out an individualized patient treatment plan. Equally important is providing information to the patient throughout the duration of the treatment. The doctor should assist the patient to create an optimized time schedule to help make the therapy less time-consuming.

**German version published in Allergologie, Vol. 34, No. 9/2011, pp. 441-446**

## Introduction 

In hyposensitization, adherence is decisive for the success or failure of therapy [16]. 

Adherence means that all therapeutic aims set by the patient and the physician are strictly followed [9]. The term compliance is considered outdated today. Compliance means that the patient has to comply with the therapy prescribed by the physician [4]; the patient has to follow the physician’s instructions passively and is the only one responsible for therapy success or failure. 

According to the current state of knowledge, the reasons for bad adherence are manifold. Physician-related factors like for example a bad bond of trust between the patient and the physician are as important as the age, gender, and economic background of the patient. Also, therapy-related factors like side effects and complicated therapeutic schemes influence therapy adherence. Furthermore, disease-specific factors that influence adherence have been identified [2], with disease severity, symptom intensity, and duration of the disease playing a major role. Particularly in long-term therapies like hyposensitization is adherence decisive for success or failure [4, 14]. 

The German Allergy and Asthma Association (Deutscher Allergie- und Asthmabund (DAAB)) in association with the Department of Medicine Statistics, Informatics and Epidemiology of the University Hospital Cologne is aiming at investigating positive and negative influencing factors for therapy adherence in hyposensitization. Following the presentation of the survey, we would like to present possible strategies for adherence improvement. 

The different treatment concepts for hyposensitization, subcutaneous immunotherapy (SCIT) [1, 7, 8] and sublingual immunotherapy (SLIT) [7, 8, 10] will be examined. Current studies have shown that in SLIT the adherence of pediatric as well as adult patients is good, although the patients have to do many things on their own [11, 12]. For this reason it is necessary to validate each procedure separately. 

## Material and methods 

Four questionnaires for various groups of patients were designed:

- Questionnaire A: Allergy patients who completed hyposensitization therapy.
- Questionnaire B: Allergy patients undergoing immunotherapy.
- Questionnaire C: Allergy patients shortly before initiation of therapy.
- Questionnaire D: Allergy patients without experience with hyposensitization.

The questionnaires were arranged in four sections. 

Part 1 of the questionnaires assessed the patient’s *knowledge of and reasons for deciding on hyposensitization.* Among other things, they were asked to rate the following needs and wishes for therapy: comprehensive information on therapy, few side effects, enduring reduction of symptoms, effectiveness evaluation by the physician, reduction of drug use, and easy integration into everyday life. The following five questions were designed to test the patient’s knowledge of hyposensitization:

- “Hyposensitization accustoms the patient to the allergen”;
- “hyposensitization makes the patient more sensitive to the allergen”;
- “side effects always occur”;
- “hyposensitization prevents allergic bronchial asthma”;
- “new allergies will develop.”

The patients had to answer these questions with “I agree” or “I do not agree”. 

In the second part, the *allergy history of the participants was assessed.*

In the third part, *data on the therapy itself were investigated.* In addition, the patient’s experience with symptomatic therapy options was inquired. 

The form of hyposensitization, the (scheduled) treatment duration, and the profile of side effects was investigated. 

The disadvantages of hyposensitization were evaluated based on the following statements: “I did not feel sufficiently informed about the therapy”; “The therapy was too time consuming”; “The therapy was difficult to integrate into everyday life”; “Side effects were frequent”; “My symptoms have not improved so far”; “I do not take less drugs so far”; “Friends and relatives have advised against the therapy.” 

The information provided by the physician and the patient’s satisfaction with the therapy were to be evaluated using a school grade scale. 

The last paragraph of the questionnaires covered *demographic data.*

The questionnaires were distributed via “AllergieMobil” (a mobile information center) [5] of the DAAB or sent to DAAB members by mail/e-mail. Furthermore, anonymous participation was possible for patients of ENT doctors. In the period March – October 2008, a total of 790 completed questionnaires were received. Descriptive statistical evaluation was carried out using the software SPSS 17.0. In addition to the evaluation of the total collective of Questionnaires A – D, a subgroup analysis was also carried out for Questionnaires A and B, where we differentiated between dosage form and therapy adherence. 

## Results 

272 patients completed Questionnaire A. 140 (69.7%) were female and 61 (30.3%) male. The mean age was 45.7 ± 14.6 years, range 6 – 85 years. SLIT was carried out in 16 patients and SCIT was carried out in 225 patients. 31 patients did not indicate the form of therapy. 

**Figure 1.**
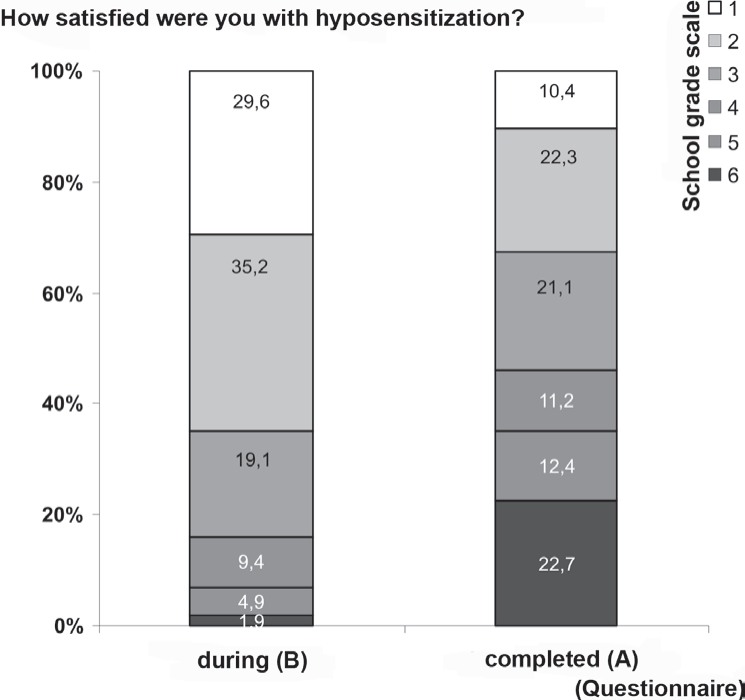
Differences in “Satisfaction with therapy” during and after therapy.

15.8% of patients indicated having discontinued therapy. The discontinuation rate was higher in women (16.8%) than in men (12.3%). 74.0% of all patients would recommend hyposensitization to their friends and family. Details on general satisfaction with the therapy are presented in Figure 1. 

**Figure 2.**
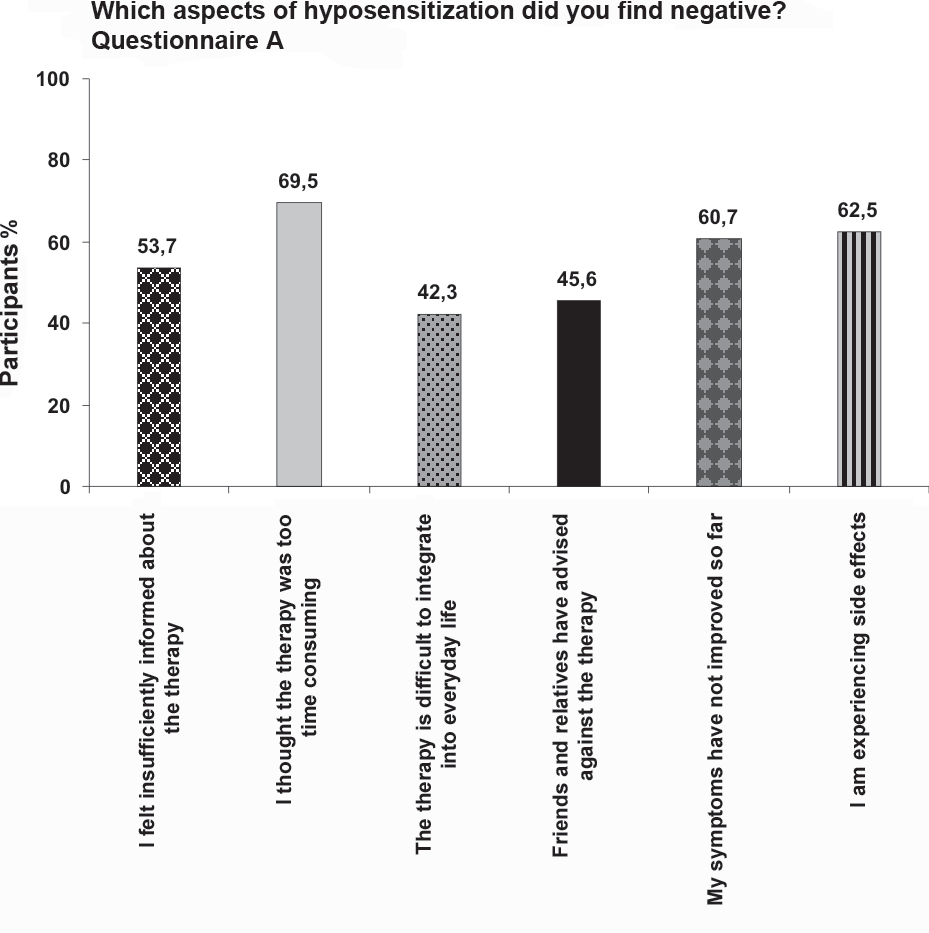
Aspects of immunotherapy that are considered disadvantageous by the patients.

Various aspects of hyposensitization were rated as disadvantageous by patients (Figure 2). 

53.7% felt insufficiently informed by their physician. This feeling can be demonstrated by the answers to the five questions on facts about hyposensitization. Not even half of the participants (47.1%) could answer all five questions correctly. 

69.5% felt that the therapy was too time consuming. Particularly for the SCIT subgroup was this a decisive factor. 72.0% of all patients perceived the time factor as a disadvantage and 42.7% said they had difficulties integrating the treatment into everyday life. On the other hand, of the patients receiving SLIT only 37.5% saw the time factor as disadvantageous and only 25% indicated the integration into everyday life to be difficult. 

In 45.6% friends and relatives advised against hyposensitization. 60.7% of patients could not detect symptom reduction and drug use was not reduced in 33.8%. 

62.5% of patients experienced side effects during the therapy. In the subgroup of participants who discontinued therapy, the percentage of patients experiencing side effects was particularly high (90.0%). In the subgroup of SCIT patients, 69.1% reported side effects, mainly local reactions (83.6%), but also circulatory complaints (20.4%), rash (17.8%), or respiratory problems (14.5%). In contrast, only 25.1% of SLIT patients indicated having suffered from side effects. 

281 patients (126 (45.5%) male and 151 (54.5%) female) completed Questionnaire B during their hyposensitization therapy. Their mean age was 36.7 ± 15.4 years (range 2 – 72 years). SLIT was carried out in 29 patients and SCIT was carried out in 238 patients. 14 patients did not indicate which form of therapy they used. At the time of the survey, 8.7% of all patients had already thought about an early discontinuation of therapy. 95.4% of all patients would recommend hyposensitization to their friends and family. In contrast to patients with previous therapy, patients with current hyposensitization tended to be very satisfied with the treatment (Figure 1). 

Nevertheless, they also named some drawbacks. 

54.8% felt they were insufficiently informed about the treatment. Despite the fact that many patients had been treated with hyposensitization for several years, only 58.4% of participants were able to answer all of the five above-mentioned test questions correctly. 

66.2% felt that the therapy was too time consuming. 51.2% of patients had not experienced any symptom improvement by the time of the survey and drug use had not been reduced in 47.0% of patients. 

63.9% of SCIT patients and 48.1% of SLIT patients suffered from side effects. At the beginning of SCIT these side effects were predominantly local reactions (97.3%), but also sneezing (12.2%), rash (8.2%), circulatory complaints (5.4%), or respiratory problems (4.8%). 

55 allergy patients completed Questionnaire C before the onset of therapy. 30 were female (55.6%) and 24 were male (44.4%). The mean age was 30 ± 14.8 years (range 3 – 69 years). 

**Figure 3.**
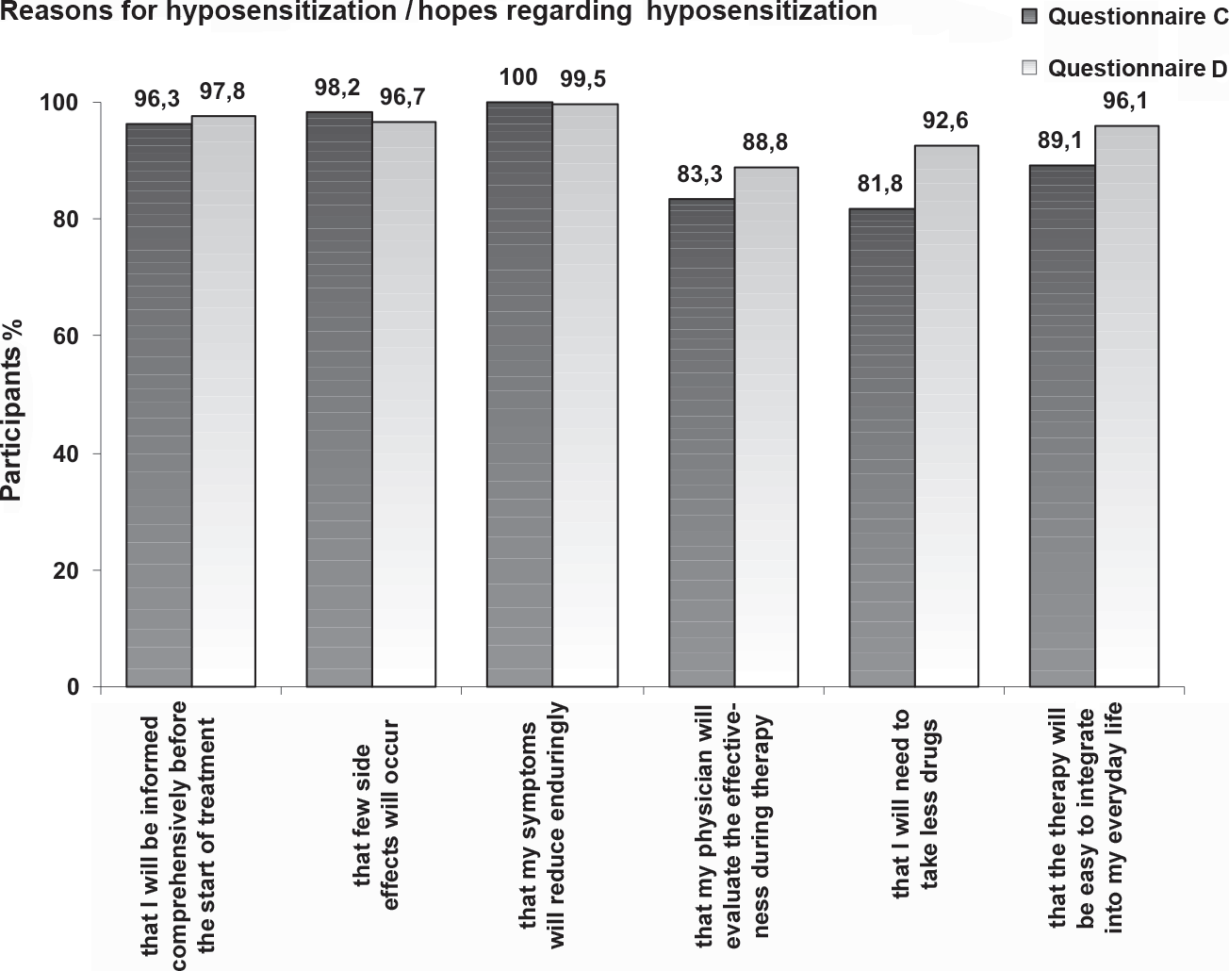
Decision criteria for therapy.

They indicated an enduring reduction of symptoms (100%), few side effects (98.2%), comprehensive information about therapy (96.3%), easy integration into everyday life (89.1%), effectiveness evaluation by their physician (83.3%), and reduction of drug use (81,8%) to be “important” or “very important” reasons for the therapy (Figure 3). Before the start of therapy the participants felt well informed (average of 2.1 on a school grade scale with 1 being “very good” and 6 being “unsatisfactory”). Nevertheless, only 47.3% of patients could answer all five test questions correctly. 

The patients had been previously treated as follows: medication as needed (69.1%), allergen avoidance (41.8%), perennial drug treatment (18.2%) and alternative medicine (9.1%). The average satisfaction with these methods was 3.0 (school grade scale). 

182 participants (135 (75.4) female, 44 (24.6%) male) completed Questionnaire D. The mean age was 42.7 ± 16.4 years (range 4 – 81 years). 

Although this questionnaire was filled in by allergy patients without previous contact with hyposensitization, 89.0% “had already heard about hyposensitization.” The patients rated their own knowledge of hyposensitization therapy with an average grade of 3.2. This could also be demonstrated by the five test questions: only 44% could answer all of them correctly. 

The patients’ hopes in regard to therapy were similar to those in Questionnaire C. Enduring reduction of symptoms (99.5%), comprehensive information on therapy (97.8%), few side effects (96.7%), easy integration into everyday life (96.1%), reduction of drug use (92.6%) and effectiveness evaluation by their physician (83.3%) were considered to be “very important” or “important” (Figure 3). 

The patients had been previously treated as follows: medication as needed (63.2%), allergen avoidance (44.0%), perennial drug treatment (24.7%) and alternative medicine (21.4%). The average satisfaction with these methods was 3.0 (school grade scale). 

## Discussion 

Successful hyposensitization requires active cooperation by the patient and particular commitment by the physician. The therapy drop-out rate in this survey was 15.8% which is relatively low compared to other studies [6]. Nevertheless, we could also identify circumstances that the patients considered particularly disadvantageous for therapy adherence: insufficient information provided by the physician, high expenditure of time (particularly for SCIT as compared to SLIT), side effects, unsatisfactory reduction of symptoms as well as deficient reduction of drug use. Similar factors influencing therapy adherence have already been reported for SCIT by Cohn and Pizzi [3] as well as by Rhodes [13]. Current research confirms the same results for SLIT [15]. 

Before the start of immunotherapy, the physician should inform the patient comprehensively and the most adequate form of hyposensitization (SCIT/SLIT) should be decided on together with the patient. This includes a detailed job-related and time-related patient history, and the patient should be asked about his/her expectations for treatment. The patient should be informed about the fact that concomitant symptomatic medication will be necessary in the beginning and that side effects are frequent. It is useful to base the individual treatment plan on the guideline for specific immunotherapy (hyposensitization) in IgE-mediated allergic diseases [7]. Standardized and – in cases of a potentially life-threatening therapy – legally admissible patient information by the manufacturer of the preparations used would be desirable. 

The physician needs to try to fulfill the patient’s needs and wishes also during the course of hyposensitization. The patient expects monitoring and information on the therapy beyond the legal requirements. The fact that at all stages only half of the patients had exact knowledge about the effects of immunotherapy shows that patients have to be much better informed. Today, a patient will not be satisfied with an “injection in passing.” Treatment success can for example be objectively assessed by nasal or conjunctival provocation testing providing the patient with a better understanding of current progress. The treatment aspects that the patients perceive as negative, above all the component “time”, can for instance be improved by special times for hyposensitization, special consultation hours for working people, and also by effective appointment making. 

Paying attention to these factors should help the physician to select appropriate patients for immunotherapy, to improve their adherence, and to reduce the still high therapy drop-out rate [2].
